# Evaluation of Advanced Field Epidemiology Training Programs in the Eastern Mediterranean Region: A Multi-Country Study

**DOI:** 10.3389/fpubh.2021.684174

**Published:** 2021-07-22

**Authors:** Mohannad Al Nsour, Yousef Khader, Haitham Bashier, Majd Alsoukhni

**Affiliations:** ^1^Global Health Development/Eastern Mediterranean Public Health Network, Amman, Jordan; ^2^Department of Public Health, Jordan University of Science and Technology, Irbid, Jordan; ^3^Center of Excellence for Applied Epidemiology, Global Health Development/Eastern Mediterranean Public Health Network, Amman, Jordan

**Keywords:** field epidemiology, public health, outbreak, surveillance, evaluation

## Abstract

Field Epidemiology Training Programs (FETPs) are competency-based training programs aiming to strengthen the epidemiologic capacity of the public health workforce. This study aimed to evaluate the impact of the advanced FETPs in the Eastern Mediterranean region (EMR) and ascertain whether the expected objectives of the programs are met. A descriptive study was conducted based on Kirkpatrick's model for evaluating training programs. Data were collected from FETP graduates and FETP technical advisers on the practices of FETP graduates, their engagement in key areas of field epidemiology, and their perceived skills and capacity to perform such activities. A total of 166 FETP graduates responded to the online survey. Almost two-thirds of FETP graduates reported that they are often engaged in managing public health surveillance systems (*n* = 119, 71.7%), analyzing the surveillance data (*n* = 116, 69.9%), training public health professionals (*n* = 113, 68.1%), investigations on and response to outbreaks (*n* = 109, 65.7%), and managing staff and resources (*n* = 106, 63.9%). However, only 28.3% reported that they are often engaged in writing scientific research articles. More than two-thirds of graduates reported that the FETP helped them to perform most of the field epidemiology activities and rate their skills as good. In conclusion, the FETP graduates in the EMR were well engaged in many field epidemiology activities including managing public health surveillance systems, surveillance data analysis, training public health professionals, and investigations on and response to outbreaks. Therefore, the FETPs should continue supporting the graduates to work toward strengthening surveillance systems and investigating outbreaks and to participate in regional and global efforts as part of the Global Health Security.

## Background

The Eastern Mediterranean region (EMR) consists of 21 countries. Many of these countries have recently experienced unrest as a result of revolutions, wars, political conflicts, massive forced displacement, and outbreaks of diseases. According to the WHO, EMR is the host to some of the biggest emergencies and protracted crises in the world. More than 29 million (58%) of the total refugees and internally displaced persons (IDPs) worldwide are from the EMR (1). With recent emergencies in the region, both displaced populations and host communities are exposed to increased public health risks. These include infectious diseases due to overcrowded living conditions, increased burden of non-communicable disease, limited access to safe water and sanitation, and changed degrees of access to primary healthcare services (2). These risks play a significant role in determining the health security of the entire region and increase the demand for public health professionals with various set of skills (3). Currently, the COVID-19 pandemic showed the need for increasing the number of individuals trained globally to prevent, detect, or respond to public health threats and the need for increasing the laboratory diagnostic testing capacity, surveillance system, and routine reporting in countries and regionally.

Field Epidemiology Training Programs (FETPs) are competency-based training programs that highly contribute to the development of national and regional health security infrastructure and the enhancement of the epidemiologic capacity of the public health workforce (4). FETP is identified as one of the key activities of the U.S. Centers for Disease Control and Prevention (CDC) in improving global health (5). Since 1980, CDC and partner organizations assisted and supported Ministries of Health (MOHs) and other public health authorities to establish FETPs in their countries (6). The training is “learning by doing,” and trainees spend more than 75% of their time in the field. They learn about investigating the outbreak, conducting studies, and training other healthcare workers (7). There are three different modalities for field epidemiology training including a 3-month basic/frontline program, 1-year intermediate FETP, and 2-year advanced FETP (4). Governmental public health workers who are responsible for public health functions at a national level of the health system are the target audience for FETPs, especially physicians, laboratory personnel, public health officers, and veterinarians.

The programs provide participants with practical experience, with minimum classroom time, and with maximum time in the field, in responding to disease outbreaks, disease surveillance, natural disasters, and other public health priorities. These programs aim at improving and strengthening the public health systems of the host country to detect, investigate, and respond quickly and effectively to public health events; establish and effectively use a robust surveillance system; develop human capacity in applied epidemiology and allied areas; and ensure that public health decisions are driven by scientific data.

Worldwide, there are 72 training programs with 14,000 graduates and 4,770 current trainees (6). In the EMR, the Eastern Mediterranean Public Health Network (EMPHNET) has helped to launch and establish several of the existing programs. Currently, there are 10 field epidemiology country training programs. Since 1989, 91 cohorts completed the training in the region with more than 700 FETP graduates (4). Most of those FETP graduates work as government officials, and many have obtained leadership positions within their national health systems (4).

Evaluation and monitoring of FETPs can be used to improve the programs and to inform decisions about future resource allocations. The evaluation helps to determine the impact of the program and how its components work toward supporting the desired outcomes. It also helps to achieve and maintain high-quality training and assure the effectiveness of the program in improving public health (8). There are some studies that evaluated the FETPs and reported the experiences and the lessons learned. These studies showed that the training programs have contributed to the development of a skilled workforce in field epidemiology (9–11). However, they indicated that further efforts are required to scale up the program.

In the EMR, studies on the evaluation of FETPs and their impacts on the healthcare systems are lacking. This evaluation is of paramount importance because the public health system in the EMR is facing many challenges, including limited resources and funding gaps, lack of or weak health information system, protracted humanitarian emergencies, fragile health systems, rapid urbanization, weak surveillance and limited laboratory diagnostic capacity, and growing frequency, duration, and scale of disease outbreaks. This study aimed to evaluate the impact of the advanced FETPs in the EMR and ascertain whether the expected objectives of the programs are met, as perceived by FETP graduates and the technical advisers of the programs. In particular, this study aimed to measure the degree to which FETP graduates applied what they learned during the training and assessed the overall perception of the technical advisers of the programs about the impact of the program on the public health system.

## Methods

### Study Design

A descriptive study was conducted during the period February 2020-May 2020. This study is based on Kirkpatrick's model for evaluating training programs (12). We adopted Kirkpatrick's model because it is a globally recognized method of evaluating the results of training and learning programs, and the model provides a logical structure and process to measure learning. When used in its entirety, it can give organizations an overall perspective of their training program and the changes that must be made. Our study focused on levels 3 and 4 of the model. Level 3 of this model is about behavior “the degree to which trainees apply what they learned during training when they are back on the job.” Level 4 is about results “the degree to which targeted outcomes occur as a result of the training and the support and accountability package” (12). The targeted outcomes were assessed from the perspectives of technical advisers. They included improved data collection on reportable diseases, improved investigations on and response to outbreaks, improved surveillance systems and surveillance reports, and improved health policies. Level 1 “the degree to which trainees find the training favorable, engaging, and relevant to their jobs” and level 2 “the degree to which trainees acquire the intended knowledge, skills, attitude, confidence, and commitment based on their participation in the training” were not assessed in this study.

### Data Collection

The data were collected using two separate online surveys/questionnaires. The first survey was delivered to advanced FETP graduates from the eight countries in the EMR that adopted this modality. Those countries include Saudi Arabia, Egypt, Jordan, Pakistan, Iraq, Morocco, Yemen, and Sudan (4). This survey was designed to assess the practices of FETP graduates asking about their involvement and engagement in key areas of field epidemiology, the extent to which FETP helped them to perform specific field epidemiology activities, and their perceived skills and capacity to perform such activities. For each question, the participants were requested to choose an appropriate response on a Likert scale. The questions covered all the competencies of the program, based on the FETP Development Handbook of the CDC (8), including epidemiology, surveillance, investigations on and response to outbreaks, research, communication, leadership, and management. The questionnaire collected information on the demographic characteristics of the participants, the highest educational degree earned, and country and year of graduation. The questionnaire was pilot tested on 10 FETP graduates, and it was revised based on their feedback. The FETP database was used to extract the emails of the graduates. The FETP database serves as a directory listing the names and career-related and contact information of FETP alumni from the EMR. The database is compiled and maintained by the Global Health Development (GHD)/EMPHNET. The database includes only 300 advanced FETP graduates with full contact information. Therefore, the questionnaire was sent by email to those graduates only. The second survey targeted the technical advisers of the programs in the selected countries who are at the level where they can observe the impact of the program. The technical advisers are responsible to teach the program courses and provide technical support to residents during field training. The survey was designed to assess the impact of the FETP on the health system and the benefit to organizations in terms of public health priorities (disease surveillance, disease outbreaks, and investigation). The questionnaire included an open-ended question to enable the respondents to make suggestions for the improvement of the FETP.

### Analysis

Data were exported to the IBM SPSS (IBM Corp, Released 2016, IBM SPSS Statistics for Windows, Version 24.0, Armonk, NY: IBM Corp.) for analysis. Data were described using percentages. The frequency distributions were presented for the main three outcome variables: engagement of FETP graduates in field epidemiology activities (often, sometimes, rarely, and never), the extent to which FETP has helped the graduates to perform field epidemiology activities (much, somewhat, little, and never), and perceived skills and capacity of the FETP graduates (good, acceptable, and poor).

## Results

### Characteristics of the Participants

A total of 166 FETP graduates from eight countries in the EMR responded to the online survey. Of those, 60 (36.1%) were women and 106 (63.9%) were men. Almost one-third (*n* = 64, 38.6%) graduated before the year 2016. Half (*n* = 84, 50.6%) of the FETP graduates continued their higher education and earned a Master or doctoral degree. The characteristics of the participants are shown in Table 1. A total of 10 FETP technical advisers have evaluated the FETPs in their countries. Six technical advisers had a doctoral degree and four had a Master degree.

**Table 1:** The characteristics of 166 Field Epidemiology Training Program (FETP) graduates in EMR.

**Variable**	***n***	**%**
**Gender**		
Female	60	36.1
Male	106	63.9
**Age (year)**		
<40	60	36.1
40–45	48	28.9
>45	58	34.9
**Graduation year**		
Before 2016	64	38.6
2016–2017	46	27.7
2018–2019	56	33.7
**Highest educational degree earned**		
Bachelor	30	18.1
Higher diploma	52	31.3
Master	55	33.1
Doctoral	29	17.5
**Country**		
Sudan	21	12.7
Egypt	30	18.1
Yemen	28	16.9
Jordan	30	18.1
Iraq	25	15.1
Pakistan	16	9.6
Morocco	12	7.2
Saudi Arabia	4	2.4

### Engagement of FETP Graduates in Field Epidemiology Activities

Almost two-thirds of FETP graduates reported that they are often engaged in activities such as managing public health surveillance systems (*n* = 119, 71.7%), analyzing the surveillance data (*n* = 116, 69.9%), training public health professionals (*n* = 113, 68.1%), investigating and responding to outbreaks (*n* = 109, 65.7%), and managing staff and resources (*n* = 106, 63.9%). A total of 121 (72.9%) participants reported they often use computers for specific applications relevant to public health practices (Table 2). The engagement of FETP graduates was the least in publishing research articles where only 28.3% reported that they are often engaged in writing scientific research articles. Moreover, less than half of the participants were often engaged in using epidemiologic methods to conduct studies that improve health program delivery, participate in public health research, and develop policy or strategy.

**Table 2:** The extent of engagement of FETP graduates in field epidemiology activities.

**Field epidemiology activities**	**Often**		**Sometimes**		**Rarely**		**Never**	
	***n***	**%**	***n***	**%**	***n***	**%**	***n***	**%**
Using computers for specific applications relevant to public health practices	121	72.9	34	20.5	8	4.8	3	1.8
Managing public health surveillance system	119	71.7	34	20.5	9	5.4	4	2.4
Analyzing the surveillance data	116	69.9	26	15.7	14	8.4	10	6.0
Training public health professionals	113	68.1	33	19.9	16	9.6	4	2.4
Outbreaks investigations and response	109	65.7	32	19.3	20	12.0	5	3.0
Managing staff and resources	106	63.9	40	24.1	14	8.4	6	3.6
Developing and delivering oral public health communications	99	59.6	44	26.5	14	8.4	9	5.4
Using laboratory resources to support public health activities	97	58.4	30	18.1	19	11.4	20	12.0
Writing public health communications	94	56.6	42	25.3	19	11.4	10	6.0
Mentoring public health professionals	91	54.8	43	25.9	19	11.4	12	7.2
Managing field projects	91	54.8	43	25.9	24	14.5	7	4.2
Developing public health interventions	89	53.6	47	28.3	22	13.3	8	4.8
Implementing public health interventions	88	53.0	44	26.5	28	16.9	6	3.6
Evaluating and prioritizing diseases or conditions of national public health concern	86	51.8	39	23.5	27	16.3	14	8.4
Using epidemiological methods to conduct studies that improve health program delivery	75	45.2	49	29.5	30	18.1	12	7.2
Participating in public health research	72	43.4	60	36.1	27	16.3	7	4.2
Policy or strategy development	70	42.2	59	35.5	25	15.1	12	7.2
Publishing scientific articles in journals	47	28.3	42	25.3	39	23.5	38	22.9

From the perspectives of technical advisers, all reported that FETP graduates are involved in managing surveillance data, developing surveillance reports, and participating in outbreak investigations to large extent. A total of four (40%) advisers reported that FETP graduates often evaluate and participate in the planning and implementation of public health interventions, and six advisers reported that they sometimes do that. When they were asked about how frequent FETP graduates use surveillance data to provide courses of action and recommendations, 60% reported always and 40% reported sometimes. Only four (40%) advisers reported the FETP graduates played a key role in regional-scale outbreaks. When they were asked about how often do FETP graduates provide informal consultations to MOH programs, five (50%) reported very often and four (40%) reported sometimes. When they were asked about how often FETP graduates use laboratory resources to support epidemiologic activities such as using laboratory data for surveillance purposes, three (30%) reported often and six (60%) reported sometimes.

### The Extent to Which Field Epidemiology Training Program Helped the Graduates to Perform Field Epidemiology Activities

More than two-thirds of graduates reported that the FETP helped them much to perform most of the field epidemiology activities listed in Table 3. However, only 76 (45.8%) graduates reported that FETP helped them much to apply simple tools for economic analysis, to publish scientific articles in journals (89, 53.6%), and to develop policies or strategies (93, 56.0%).

**Table 3:** The extent to which FETP helped the graduates to perform field epidemiology activities.

**Field epidemiology activities**	**Much**		**Somewhat**		**Little**		**Never**	
	***n***	**%**	***n***	**%**	***n***	**%**	***n***	**%**
Outbreaks investigations and response	132	79.5	25	15.1	4	2.4	5	3.0
Managing public health surveillance system	130	78.3	22	13.3	5	3.0	7	4.2
Analyzing the surveillance data	127	76.5	28	16.9	7	4.2	4	2.4
Developing a public health intervention	123	74.1	27	16.3	5	3.0	11	6.6
Mentoring public health professionals	122	73.5	26	15.7	6	3.6	12	7.2
Training public health professionals	120	72.3	34	20.5	8	4.8	4	2.4
Implementing public health interventions	120	72.3	30	18.1	5	3.0	11	6.6
Using computers for specific applications relevant to public health practices	120	72.3	27	16.3	8	4.8	11	6.6
Evaluating and prioritizing the importance of diseases or conditions of national public health concern	116	69.9	30	18.1	6	3.6	14	8.4
Managing field projects	115	69.3	35	21.1	5	3.0	11	6.6
Using epidemiological methods to conduct studies that improve health program delivery	114	68.7	36	21.7	4	2.4	12	7.2
Writing public health communications	114	68.7	37	22.3	7	4.2	8	4.8
Participating in public health research	112	67.5	42	25.3	7	4.2	5	3.0
Developing and delivering oral public health communications	112	67.5	38	22.9	6	3.6	10	6.0
Managing staff and resources	104	62.7	37	22.3	12	7.2	13	7.8
Policy or strategy development	93	56.0	47	28.3	9	5.4	17	10.2
Using laboratory resources to support public health activities	92	55.4	42	25.3	17	10.2	14	8.4
Publishing scientific articles in journals	89	53.6	41	24.7	16	9.6	20	12.0
Applying simple tools for economic analysis	76	45.8	52	31.3	20	12.0	18	10.8

### Skills and Capacity of the Field Epidemiology Training Program Graduates

More than two-thirds of the FETP graduates rated their skills to conduct many field epidemiology activities as good (Table 4). However, much smaller percentages of the graduates reported that their skills are good in applying simple tools for economic analysis (65, 39.2%) and writing scientific research articles (67, 40.4%).

**Table 4:** Self-evaluation of FETP graduates of their skills in performing field epidemiology activities.

**Field epidemiology activities**	**Good**		**Acceptable**		**Poor**	
	***n***	**%**	***n***	**%**	***n***	**%**
Training public health professionals	128	77.1	30	18.1	7	4.2
Managing staff and resources	121	72.9	36	21.7	9	5.4
Outbreaks investigations and response	120	72.3	37	22.3	9	5.4
Managing public health surveillance system	119	71.7	34	20.5	13	7.8
Mentoring public health professionals	116	69.9	39	23.5	11	6.6
Managing field projects	115	69.3	38	22.9	12	7.2
Developing a public health intervention	114	68.7	40	24.1	12	7.2
Developing and delivering oral public health communications	111	66.9	40	24.1	15	9.0
Using computers for specific applications relevant to public health practices	111	66.9	43	25.9	12	7.2
Evaluating and prioritizing the importance of diseases or conditions of national public health concern	111	66.9	48	28.9	7	4.2
Implementing public health interventions	110	66.3	45	27.1	11	6.6
Analyzing the surveillance data	109	65.7	43	25.9	14	8.4
Writing public health communications	103	62.0	53	31.9	10	6.0
Participating in public health research	102	61.4	50	30.1	14	8.4
Using epidemiological methods to conduct studies that improve health program delivery	96	57.8	52	31.3	17	10.2
Policy or strategy development	94	56.6	48	28.9	23	13.9
Using laboratory resources to support public health activities	93	56.0	44	26.5	29	17.5
Publishing scientific articles in journals	67	40.4	53	31.9	45	27.1
Applying simple tools for economic analysis	65	39.2	54	32.5	47	28.3

Almost all technical advisers stated that the capacity of FETP graduates is very good, and they contributed to strengthening the public health system, improving the surveillance system, developing the protocols and guidelines, and improving health policies. Some advisers reported that FETP graduates are now placed at senior level decision-making positions and have started to make a very positive impact on improving the health system. Moreover, they reported that the confidence of the governments in the capacities of FETP graduated has improved manifold and the governments now rely on FETP graduates for outbreak investigation, response, and surveillance activities. Although the capacities of the FETP graduates are reported to be good, some advisers reported that they are facing difficulties in showing impact due to limited financial and human resources and war and siege.

### The Impact of the Field Epidemiology Training Program From the Perspectives of Technical Advisers

Five advisers (50%) reported that the data collection on reportable diseases has improved much in their countries since the establishment of FETP, and the rest reported that it has been somewhat improved. A total of seven (70%) technical advisers reported that the FETP has improved the investigations on and response to outbreaks in their countries to a large extent, and 30% reported that they are somewhat improved. All reported that FETP graduates contributed significantly to improvements in surveillance systems (90%). A total of four (40%) advisers reported that FETP contributed much and that five (50%) contributed somewhat to regular national reports such as providing articles, editing articles, and presenting surveillance data. When they were asked about how much the FETP improved health policies and contributed to the strengthening of health systems in their countries, 40% reported much and 50% reported somewhat.

### Suggestions for the Improvement Field Epidemiology Training Program From the Perspectives of Technical Advisers

Most of the technical advisers reported that there is a need for strategies to ensure the retention of FETP graduates by placing them in proper positions with good financial incentives. Some suggested that the FETP certificate should be upgraded to a scientific degree. Moreover, they recommended increasing the capacity of the programs to train more people and working on accreditation, updating the curriculum, setting a mandatory requirement for graduation, and documenting the activities and the impacts of FETP graduates.

## Discussion

Periodic evaluation of public health training programs is essential to ensure that the programs are achieving their intended outcomes and impacts. The effectiveness of the FETPs can be demonstrated by assessing the competencies of the graduates and their involvement in providing public health services (13). This study assessed the contribution of FETPs to the improvement of public health services at the country level.

A total of 166 advanced FETP graduates from eight EMR countries adopted this modality of training. Of the respondents, one-third were women. This is consistent with the women: men ratio among total advanced FETP graduates in the EMR. This study clearly demonstrated that FETPs in the EMR have met the goal of preparing graduates to implement public health and main field epidemiology measures and activities. There was a consensus between FETP graduates and technical advisers on the fact that FETP graduates in the EMR are well-engaged in many field epidemiology activities including managing public health surveillance systems and surveillance data analysis, training public health professionals, and investigating and responding to outbreaks. Their impact can be most apparent in situations of conflicts and crises that disrupt public health systems and especially within the current shortage of resources. Several examples have clearly shown the success of FETP in responding to emergencies and disasters (9–11, 13–20). During the outbreak of the Middle East respiratory syndrome (MERS) in 2014 in Saudi Arabia, FETP graduates tackled numerous issues, including but not limited to, related to redesigning the system to enable simultaneous real-time electronic reporting of suspected and confirmed cases to public health professionals who needed to take essential control and preventive actions on new cases (16). FETP graduates are working nowadays in different ways to fight the COVID-19 pandemic as they are actively participating in surveillance and screening at airports and other ports of entry, developing communication materials and guidelines, and sharing information to health professionals and to the public (18). FETPs in the EMR showed success in building the epidemiologic capacity for the public health workforce, improving the surveillance systems of the countries, and strengthening the health systems (14, 16).

However, this study showed that only small percentages of FETP graduates were engaged in applying tools for economic analysis, publishing scientific articles in journals, and developing policies or strategies. The low level of engagement in these activities is explained by inadequate skills in these areas as reported by FETP graduates. A relatively small percentage of the graduates reported that their skills are good in applying simple tools for economic analysis (39.2%) and writing scientific research articles (40.4%). Another reason for this low level of engagement is that MOHs do not realize the capacity of FETP graduates to perform these functions. Although that hundreds of outbreaks were investigated in the past years in the EMR, hundreds of studies were conducted, and many public health problems were investigated, only a small percentage of FETP graduates managed to publish their findings in international journals. This makes many achievements and success stories in public health in the region invisible and the contribution to the science and practice of epidemiology very minimal. Therefore, it is essential that the FETPs should invest more in building the capacity in this area. Supervisors and mentors need to provide FETP residents with tailored, flexible, and regular support to produce high-quality, timely, and relevant research studies.

Moreover, there is a need to enhance the capacities of trainees in the area of health economics with a focus on essential tools such as cost-effectiveness analysis, cost-benefit analysis, and cost-utility analysis. In general, there is a lack of expertise in using and applying economic tools in public health interventions in the region. Therefore, these competencies are essential to be developed and whenever possible, both economic and clinical data should be gathered in each country. The aforementioned competencies and skills can be considered in the future FETP programs to provide a holistic experience for all FETPs.

Technical advisers reported the positive impact of FETP on data collection on reportable diseases, investigations on and response to outbreaks, and surveillance systems in their countries. This finding validates the responses of the graduates regarding their engagement in field epidemiology activities and self-rating of their skills. According to the technical advisers of FETPs, the FETP graduates did not play a key role in regional-scale outbreaks. The current COVID-19 pandemic and previous outbreaks such as MERS, Swine flu (H1N1), and Ebola have demonstrated that while the outbreak may originate in one place, it can quickly spread to other parts of the world. Therefore, the FETPs should be able to mobilize resources to respond to public health emergencies. Hence, EMPHNET proposed the establishment of an FETP Residents' Exchange Program (FETP-REP) to provide the FETP residents in the region an opportunity to join FETP programs in other countries for a specific period for the purpose of gaining new experiences. This will help to build the field capacity of the residents in new public health threats not usually encountered in their home countries, exchange experiences in managing and running FETP programs, extend the regional network of public health specialists and field epidemiologists, strengthen the regional emergency response mechanism, and enhance coordination among MOHs in the region. Moreover, EMPHNET had already undertaken some activities and initiatives to connect FETP graduates (website, EpiShares, social media channels, or FETP ambassador program). Although the use of these channels has been effective to some extent, these platforms on their own will not serve as a full-fledged community of practice because they are simply missing the human element of interaction. Therefore, an alumni association is proposed to fill this gap and to bring together a dynamic community of FETP graduates that is motivated and dedicated to improving health in the region and to serve as a space for discussion groups and networking opportunities among FETP graduates to share experiences, interests, and needs in areas of public health relevant to the region.

One of the strengths of this study is that we assessed level 3 “the degree of applying what learned” and level 4 “the degree to which outcomes occur as a result of the training” of Kirkpatrick's model in evaluating FETP training programs (12). Although Kirkpatrick's model was primarily designed for industry, it is often used to structure trainings for health professionals (21, 22). The popularity of this model can be traced to several factors. It mainly provides a straightforward systematic way for evaluating training outcomes and provides information that can be used to assess the extent to which training programs have achieved certain objectives (23). Another strength is that the evaluation was based on information from two sources including the FETP graduates and advisers of the programs who are within the health system at a level where they can observe the impact of the program. Our results were derived from an online survey with all its potential strengths and limitations. The survey was anonymous, and thus, it is very likely that participants gave correct answers without being afraid of exposing their identity. Also, they were not under any pressure to give “desirable answers” to the survey questions. This study has many limitations. This study examined the extent to which graduates are involved in related activities and how they perceive their skill levels for these activities, but this did not include formal evaluation of program competencies and objective assessment of the skills of the graduates. On one hand, the sample size of our evaluation was limited by the number of graduates whose complete contact information was available. On the other hand, we made every effort to increase the response rate by sending the graduates three reminders to respond to the questionnaire and by asking the technical advisers to encourage graduates to participate in this study. However, only 55.3% of invited graduates had responded to the survey. One should consider this limitation when interpreting the findings of this study. It worth mentioning that the gender and age distribution in the sample reflected that of all FETP graduates. However, graduates from Saudi Arabia were underrepresented in the sample. Another limitation is that we assessed the perception of technical advisers regarding the impact of the FETP. Future in-depth studies are needed to quantitatively assess the impact of indicators such as improved data collection on reportable diseases, improved outbreak investigation and response, improved surveillance systems, and improved health policies. Another limitation is the possibility of reporting favorable responses to some questions related to the extent to which FETP helped graduates to perform the field epidemiology activities. For example, the persons who have undergone the training in outbreak investigation are expected to tell that the training was useful in investigating outbreaks.

## Conclusions

In conclusion, the FETP graduates in the EMR were well-engaged in many field epidemiology activities including managing public health surveillance systems, surveillance data analysis, training public health professionals, and investigating and responding to outbreaks. Therefore, the FETPs should continue supporting the graduates to work toward strengthening surveillance systems and investigating outbreaks and to participate in regional and global efforts as part of the Global Health Security. The establishment of new FETPs in other countries of the region should be supported to improve public health in all countries of the region.

## Data Availability Statement

The raw data supporting the conclusions of this article will be made available by the authors, without undue reservation.

## Ethics Statement

The studies involving human participants were reviewed and approved by Institutional Review Board at Jordan University of Science and Technology. Written informed consent for participation was not required for this study in accordance with the national legislation and the institutional requirements.

## Author Contributions

MoA, MaA, HB, and YK contributed to study design, data analysis, and writing the manuscript. MaA contributed to data collection and revision of the manuscript. All authors have read and approved the manuscript.

## Conflict of Interest

The authors declare that the research was conducted in the absence of any commercial or financial relationships that could be construed as a potential conflict of interest.
